# Investigation of Markedly Elevated Liver Enzymes With Serendipitous Underlying Wilson’s Disease With Chronic Alcohol Abuse

**DOI:** 10.7759/cureus.59025

**Published:** 2024-04-25

**Authors:** Khiet T Nguyen, Dat D Nguyen, Leidhy Montecinos, Pwint P Hlaing, Samridhi Khatri

**Affiliations:** 1 Medicine, Interfaith Medical Center, New York, USA; 2 Interventional Cardiology, Gia Dinh People's Hospital, Ho Chi Minh, VNM; 3 Internal Medicine, Interfaith Medical Center, New York, USA; 4 Internal Medicine, Eastern Maine Medical Center, Bangor, USA

**Keywords:** elevated triglycerides, alcoholic hepatitis, hemochromatosis, wilson’s disease, markedly elevated liver enzymes

## Abstract

Acute hepatitis can result from a wide variety of noninfectious causes that include, but are not limited to, drugs (drug-induced hepatitis), alcohol (alcoholic hepatitis), immunologic (autoimmune hepatitis, primary biliary cholangitis), or as a result of indirect insult secondary to biliary tract dysfunction (cholestatic hepatitis), pregnancy-related liver dysfunction, shock, or metastatic disease. In clinical settings, these causes are not uncommon to overlap with each other or are masked by obviously visible causes in medical history. We reported our scenario of a patient who has a heavy history of alcohol use and presented with alcohol withdrawal symptoms and a marked elevation of liver enzymes. Interestingly, further investigations suggested Wilson's disease could be an underlying culprit of acute hepatitis in this patient. This case again emphasized that Wilson's disease can be masked under multiple causes and various scenarios, which alerts clinicians that a broad approach should be made for every case of acute hepatitis.

## Introduction

Wilson's disease is an autosomal recessive inherited disorder of copper metabolism with a prevalence of approximately 1:30,000 [1]. Clinical scenarios for Wilson's disease could vary from acute hepatitis failure to chronic hepatitis to cirrhosis [2]. The diagnosis of Willson's disease, which causes elevated transaminase, could be challenging when other apparent causes, such as alcohol abuse, hepatitis virus infection, or an iron profile favored for hemochromatosis, could masquerade as the actual cause. Due to the non-specific symptoms of Wilson's disease during its early stages, should physicians consider Wilson's disease as something that must be investigated at a young age in order to warrant early treatment or liver transplant screening criteria?

We report a case of a 26-year-old female who heavily consumed alcohol daily for more than eight years and initially presented severely elevated transaminitis with alcohol withdrawal symptoms. Subsequent examination indicated that Wilson's disease could be the underlying cause of this scenario.

## Case presentation

A 26-year-old female with a history of chronic alcohol abuse, consuming one bottle of vodka and half a liter of whisky daily since the age of 18, presented to the emergency department requesting alcohol detox due to poor appetite and weakness. The patient has been experiencing not feeling well and showed some tremors with nausea and vomiting, generalized body aches, and general weakness. The patient consumed alcohol the day before admission in an attempt to control the withdrawal symptoms.

Two weeks before this admission, the patient completed 10 days of alcohol detox in another hospital. During the physical exam, the patient was alert, awake, and oriented. The physical examination was unremarkable, and there was mild epigastric tenderness. The Clinical Institute Withdrawal Assessment for Alcohol (CIWA-Ar) score of the patient is 8, which indicates minimal to mild withdrawal.

The family history is remarkable for liver diseases, but it is unclear in detail.

The blood test showed severely elevated liver enzymes with aspartate aminotransferase (AST) level of >2400 mg/dL, alanine transaminase (ALT) of 2109 mg/dL, international normalized ratio (INR) of 1.58, alkaline phosphatase (ALP) of 103 mg/dL, total bilirubin level of 1.4 mg/dL, direct bilirubin of 0.66 mg/dL, and fibrinogen of 103 mg/dL. Serum alcohol was 280 mg/dL. The results are described in Table 1.

**Table 1:** Laboratory results of the patient on admission AST: aspartate aminotransferase; ALT: alanine transaminase; ALP: alkaline phosphatase; PT/aPTT/INR: partial thromboplastin/activated partial thromboplastin time/international normalized ratio; GGT: gamma-glutamyl transferase; WBC: white blood cell; RBC: red blood cell; HBG: hemoglobin; HCT: hematocrit; MCV: mean corpuscular volume; MCH: mean corpuscular hemoglobin; MCHC: mean corpuscular hemoglobin concentration; RDW: red cell distribution width; MPV: mean platelet volume

Laboratory Parameters	Patient’s Value	Reference Range
AST	>2400 mg/dL	7-52 U/L
ALT	2109 mg/dL	13-39 U/L
ALP	103 mg/dL	34-104 U/L
Total bilirubin	1.4 mg/dL	0.3-1.0 mg/dL
Direct bilirubin	0.66 mg/dL	0.03-0.18 mg/dL
PT/aPTT/INR	19/32/1.58	9.8-13.4 sec/24.9-35.9 sec/0.85-1.15
GGT	>1200 mg/dL	9-64 U/L
Serum alcohol	280 mg/dL	<10 mg/dL
NH_3_	106 µmol/L	16-53 µmol/L
Lipase	150 (H) U/L	11-83 U/L
WBC	4.9x10^3^/µL	4.5-11.0x10^3^/µL
RBC	3.44(L)x10^6^/µL	3.8-5.3x10^6^/µL
HBG	12.4 g/dL	11.0-15.0 g/dL
HCT	34.8% (L)	35%-46%
MCV	101.2 (H) fL	80-100 fL
MCH	36.2 (H) pg	26.0-33.0 pg
MCHC	35.7 g/dL	30.5-36.0 g/dL
RDW	13.6%	11.5%-15.1%
MPV	6.5 (L) fL	NA
Platelets	407 (H)x10^3^/µL	130-400x10^3^/µL

The CT scan of the abdomen was unremarkable except for a diffuse thickening of the descending colon.

Although the patient has a significant medical history related to alcohol use, other causes were investigated for severe acute hepatitis, including hepatitis viruses, hemochromatosis, autoimmune diseases, and Wilson's disease. The results showed a ferritin level of 2331 mg/dL, transferrin level of 99%, negative smooth muscle antibody, negative hepatitis A, B, and C, triglyceride level of 950 mg/dL, alpha 1 antitrypsin level of 114 mg/dL, ceruloplasmin level of 9.6 mg/dL, negative antinuclear antibodies (ANA), GGT of >1200 mg/dL, negative cytomegalovirus (CMV) IgM, positive IGG, and negative Epstein-Barr virus (EBV) IgM/IgG. Additionally, the results showed that the patient is negative for liver kidney microsomal Ab, mitochondrial Ab, and hereditary hemochromatosis DNA screening. The detailed lab results are described in Table 2 below.

**Table 2:** The laboratory results of possible causes of acute hepatitis CMV: cytomegalovirus; EBV: Epstein-Barr virus; ANA: antinuclear antibodies

Laboratory Parameters	Patient’s Value	Reference Range
Ceruloplasmin	9.6 mg/dL	19.0-39.0 mg/dL
Ferritin	2331 mg/dL	11.00-306.80 ng/mL
Hereditary hemochromatosis DNA screening	Undetected	NA
Transferrin saturation	99%	NA
Anti-smooth muscle Ab	Negative	0-19 Units
Liver kidney microsomal Ab	Negative	0.0-20.0 Units
Hepatitis A	Non-reactive	NA
Hepatitis B	Non-reactive	NA
Hepatitis C	Non-reactive	NA
Hepatitis E	Non-reactive	NA
CMV	IgM negative/IGG positive	NA
EBV	IgM/IgG negative	NA
ANA	Negative	NA

The patient was managed with N-acetylcysteine (NAC) infusion protocol for non-acetaminophen hepatotoxicity with supportive care.

The ceruloplasmin result was measured at 9.6 mg/dL, indicating a strong likelihood of Wilson's disease. The patient left against medical advice after being admitted for one day in the hospital.

Other workups failed to establish a conclusive diagnosis of Wilson's disease. Subsequently, the patient was contacted by phone to convey the recommendations for further investigation of Wilson's disease.

## Discussion

Wilson's disease is a rare condition with abnormalities in copper metabolism that manifest commonly in liver and neurological functions. The range of clinical illustrations of Wilson's disease varies from fulminant hepatic failure with hemolytic anemia to chronic hepatic disease. In our case, although this patient had an extensive history of alcohol abuse since being a teenager, a markedly elevated transaminase of > 15 times the upper limit warrants further investigation for overlap of underlying conditions [3,4].

Alcoholic hepatitis commonly presents with mildly elevated ALT levels of <300 UI/L and rarely >500 IU/L. Additionally, the AST:ALT ratio is typically >2. In situations where the ALT level is >500 IU/L, even if the AST:ALT ratio is >2, other etiologies should be explored [4]. Hepatitis viruses, including HAV, HBV, HCV, HEV, EBV, and CMV, were excluded from the results. Autoimmune diseases were investigated with extensive autoimmune markers, but no evidence of these diseases was found.

In addition to that, in our case, a markedly increasing ferritin level and iron saturation of >99% would preliminarily suggest hemochromatosis. However, this was later disproven through a negative HFE gene mutation. This phenomenon can be explained by the Coombs-negative hemolysis of Wilson’s disease outburn or in fulminate hepatic failure with an acute inflammatory phase. There are several reports about iron overload in Wilson’s disease, which leads to potential secondary hemochromatosis [5].

Interestingly, a remarkably elevated triglyceride level of >950 mg/dL in this case raises the question about lipid metabolism or, more specifically, triglyceride altered in Wilson's disease itself or the result of acute hepatic injury. Further investigation may be required to explore this phenomenon in order to develop additional strategies for managing fulminate hepatic failure induced by Wilson's disease, as most conventional medications typically reduce triglyceride levels in cases of acute hepatic failure, except omega-3, insulin, and heparin infusions. However, there is no standard guideline for such.

In this case, despite lacking other signs such as Kayser-Fleischer rings, 24-hour urinary copper, liver copper level, or multiple mutations in the *ATP7B* gene, having a ceruloplasmin level lower than 10 mg/dL showed a high level of specificity (100%) and positive predictive value (100%) in the scoring system for diagnosing Wilson's disease. Further investigations can yield a conclusive diagnosis, which was limited in this case due to the patient's decision to leave against medical advice. However, based on this article, the main idea conveyed is the importance of adopting a broad approach to acute hepatitis [6]. Implementing this practice would lead to significant benefits for the patient and contribute to the reduction of preventable complications associated with cirrhosis in the long term [7].

## Conclusions

It is not justified to attribute acute hepatitis with markedly elevated liver enzymes solely to a history of extensive alcoholism. Instead, it would be beneficial to investigate other potential causes, such as Wilson's disease and hemochromatosis. By doing so, we can intervene in the entire pathological process, improve the outcome dramatically, and prevent avoidable complications in cirrhosis.
